# GastroMalign: Vision Transformer-Based Framework for Early Detection and Malignancy-Risk Stratification for High-Risk Gastrointestinal Lesions

**DOI:** 10.3390/jcm15072701

**Published:** 2026-04-02

**Authors:** Sri Harsha Boppana, Sachin Sravan Kumar Komati, Medha Sharath, Aditya Chandrashekar, Gautam Maddineni, Raja Chandra Chakinala, Pradeep Yarra, C. David Mintz

**Affiliations:** 1 Department of Internal Medicine, Nassau University Medical Center, East Meadow, NY 11554, USA; 2 Department of Computer Science, Florida International University, Miami, FL 33199, USA; 3 Department of Internal Medicine, Bangalore Medical College and Research Institute, Bangalore 560002, Indiaadityac002@gmail.com (A.C.); 4 Department of Gastroenterology & Hepatology, Creighton University School of Medicine, Omaha, NE 68124, USA; 5 Department of Gastroenterology, Guthrie Robert Packer Hospital, Sayre, PA 18840, USA; 6 Division of Gastroenterology, The University of Texas Health Science Center at San Antonio, San Antonio, TX 78229, USA; yarra@uthscsa.edu; 7 Department of Anesthesiology & Critical Care Medicine, Johns Hopkins School of Medicine, Baltimore, MD 21205, USA

**Keywords:** endoscopy, machine learning, vision transformers, convolutional neural network, prognosis, risk stratification

## Abstract

**Background**: Current artificial intelligence (AI) systems in gastrointestinal (GI) endoscopy primarily emphasize binary detection or static classification, providing limited support for the graded assessment of malignant potential that underpins clinical decision-making. We developed GastroMalign, a transformer-based framework designed to stratify GI lesions according to ordinal disease severity while maintaining clinical interpretability, addressing this unmet need in endoscopic risk assessment. **Methods**: This retrospective development and validation study used the publicly available GastroVision dataset, comprising 8000 de-identified endoscopic still images from the upper and lower gastrointestinal tract, including the esophagus, stomach, duodenum, colon, rectum, and terminal ileum. GastroMalign integrates a Vision Transformer (ViT) encoder with a Sequential Feature Learner that explicitly models ordinal disease severity along a benign-to-malignant spectrum. The framework produces both categorical risk classification and a continuous malignancy risk score. Images were stratified into training (80%), validation (10%), and test (10%) sets. Performance was compared with convolutional neural network (CNN) baselines and a Swin Transformer. Interpretability was assessed using Score-CAM visualizations reviewed by blinded expert endoscopists. **Results**: On the held-out test set (n = 800 images), GastroMalign achieved an overall accuracy of 80.06%, precision of 79.65%, recall of 80.06%, and F1-score of 79.17%, with a micro-averaged AUC of 0.98. In comparison, ResNet-50 and DenseNet-121 achieved accuracies of 32.42% and 36.77%, respectively, while the Swin Transformer achieved 60.56% accuracy (AUC = 0.93). Ablation analyses demonstrated a 17% absolute reduction in High-Risk lesion recall when the progression-aware module was removed. Continuous malignancy risk scores increased monotonically across ordinal classes, with mean values < 0.18 for Benign and >0.72 for High-Risk/Malignant lesions. Score-CAM visualizations demonstrated 92% overlap with clinician-annotated lesion regions. **Conclusions**: GastroMalign delivers an interpretable, progression-aware AI framework for GI lesion risk stratification that outperforms existing CNN- and transformer-based models. Clinically, GastroMalign is intended as an adjunct decision-support tool during endoscopic review to standardize lesion risk stratification (benign to malignant spectrum), support management decisions (biopsy vs. resection vs. surveillance), and reduce operator-dependent variability by pairing ordinal risk outputs with interpretable visual explanations.

## 1. Introduction

Colorectal cancer (CRC) remains the second leading cause of cancer-related mortality globally, and its global burden continues to rise [1]. CRC is also among the most preventable malignancies because detection and removal of precursor lesions can avert cancer development [1,2]. Based on current global incidence trends, the number of new CRC cases is projected to reach 2.36 million in 2050 [2]. Effective screening not only reduces CRC incidence and mortality but also decreases treatment-related morbidity and downstream healthcare utilization by shifting care toward prevention and early-stage intervention rather than late-stage oncologic therapy [3,4,5,6]. Despite these benefits, accurate interpretation of endoscopic findings is highly operator-dependent, with diagnostic performance often influenced by clinician experience, procedural conditions, sedation techniques, and device specifications [7,8].

To tackle these limitations, deep learning technology has increasingly been explored as decision-support tools for endoscopic image interpretation [9,10,11]. Among these, convolutional neural networks (CNNs) have driven major advances in medical image analysis and have demonstrated improved diagnostic accuracy in colonoscopy across multiple studies [9,10,11,12,13,14,15,16]. However, early neoplastic changes often manifest as subtle, spatially distributed architectural or vascular abnormalities rather than sharply demarcated lesions, making them difficult to detect using conventional image analysis approaches [14]. While CNNs have demonstrated promising results in endoscopic image classification and polyp detection [10,13], their reliance on local receptive fields can limit their ability to capture long-range spatial dependencies that are critical for accurate lesion grading and risk stratification [14,17,18].

Recent advances in transformer-based architectures, particularly Vision Transformers (ViTs), have shown strong performance in medical imaging tasks, in some settings outperforming traditional CNNs [14,19,20,21]. By modeling relationships across the entire image, ViTs are better suited to capture the complex morphological patterns relevant to lesion characterization. However, most existing deep-learning approaches for GI colonoscopy focus primarily on binary detection or static multi-class classification, and offer limited insight into lesion evolution or future malignant transformation [14]. Without explicit modeling of disease severity along a clinically meaningful spectrum, the translational utility of these systems remains constrained.

To address these challenges, we developed GastroMalign, a transformer-based deep learning framework designed for risk stratification and assessment in endoscopic imaging. GastroMalign classifies lesions into four clinically relevant malignancy risk categories (Benign, Low-Risk, High-Risk, and Malignant) and generates a complementary continuous malignancy risk score derived from the same severity-aware representation, and yields interpretable outputs that support clinician decision-making. The performance of GastroMalign was evaluated against several baseline models, including ResNet-50, DenseNet-121, a custom CNN architecture, and the Swin Transformer, to assess the added value of the proposed architecture. In addition, Score-CAM-based interpretability was incorporated to provide visual explanations that align closely with clinician-annotated lesion regions, thereby supporting transparency and clinical trust.

## 2. Methods

### 2.1. Study Design and Dataset

This was a retrospective development and validation study using the publicly available GastroVision dataset distributed under a Creative Commons Attribution 4.0 International (CC BY 4.0) license [22]. GastroVision is a public, multicenter, open-access endoscopy image dataset containing 8000 de-identified still images across 27 labeled classes from the upper and lower gastrointestinal tract, including normal findings, anatomical landmarks, pathological lesions, and selected therapeutic intervention-related views and was acquired at two centers (Bærum Hospital, Norway; Karolinska University Hospital, Sweden) using standard endoscopy systems (Olympus, Tokyo, Japan and Pentax, Tokyo, Japan). The dataset was acquired using Olympus and Pentax endoscopy systems. Most images were obtained with white-light imaging, and a smaller subset was acquired using narrow-band imaging. The public dataset documentation does not provide structured metadata on Pentax-specific enhancement submodes such as I-SCAN; therefore, these were not analyzed as a separate imaging category. Dataset labels were used as provided by the GastroVision release; per the dataset documentation, labeling and verification involved experienced gastrointestinal endoscopists together with a junior physician and computational scientists. No patient identifiers were available to the study team, and images were used in de-identified form without access to re-identification keys or protected health information.

Images were screened for both completeness and usability before partitioning. Completeness was defined as the presence of a valid image file, an associated ground-truth label, and an interpretable single-field endoscopic view of the target mucosal region without major truncation. Usability was defined as technical adequacy for visual assessment, including sufficient focus, illumination, contrast, and visible mucosal detail to permit lesion interpretation. Images were excluded if they lacked a ground-truth label, contained unreadable or corrupted files, or had insufficient visual quality for interpretation, including severe blur, poor illumination, or obscuration of the region of interest. Each image corresponds to a single-field endoscopic view of the upper or lower GI tract and was annotated independently by two board-certified gastroenterologists. A senior gastroenterologist adjudicated discordant labels to obtain the final ground truth. Images were excluded if they lacked ground-truth labels, contained unreadable or corrupted files, or had insufficient visual content for interpretation (e.g., out-of-focus frames). No imputation was performed. All exclusions were applied before stratified train/validation/test partitioning to prevent information leakage. The publicly released dataset does not provide procedure-type granularity (e.g., colonoscopy vs. flexible sigmoidoscopy) for lower GI images, nor does it include endoscopist-level metadata such as years of experience, trainee involvement, or withdrawal time. Accordingly, we treated all images as endoscopic still frames as provided and reported this lack of procedural metadata as a limitation for generalizability analyses.

**Figure 1:**
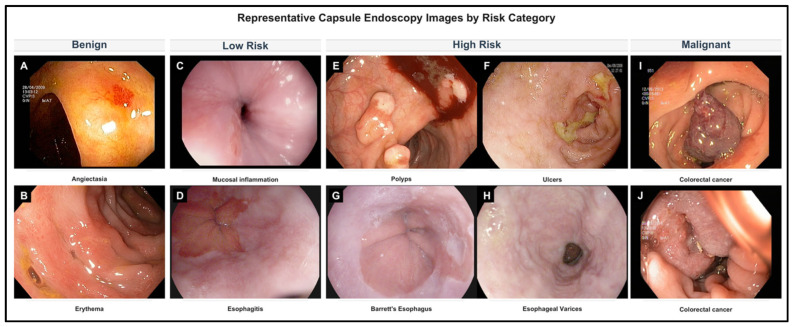
Representative examples of benign (**A**,**B**), low-risk (**C**,**D**), high-risk (**E**–**H**), and malignant (**I**,**J**) lesions in the GastroVision dataset. Representative examples of benign, low-risk, high-risk, and malignant categories derived from the GastroVision dataset. Images are de-identified endoscopic still frames from the original GastroVision release (CC BY 4.0) and illustrate typical appearances included in model training after mapping the dataset’s original labels to four malignancy-risk strata.

For this study, original GastroVision labels were mapped into four study-specific strata: Benign, Low-Risk, High-Risk, and Malignant. This mapping was based on expected malignant potential and clinical management implications as judged by the study team, and was intended to create an ordinal risk-stratification framework for model development. It should not be interpreted as a replacement for established disease-specific endoscopic or histopathologic classification systems. Lesions were grouped into four clinically relevant risk strata: Benign (e.g., normal mucosa, diverticula), Low-Risk (e.g., non-dysplastic polyps, Barrett’s esophagus without dysplasia), High-Risk (e.g., dysplastic lesions, high-risk varices), and Malignant (e.g., colorectal carcinoma, ulcerated masses) (Figure 1). Images with ambiguous or mixed pathology labels were excluded. The final dataset retained balanced representation across the four target categories.

**Figure 2:**
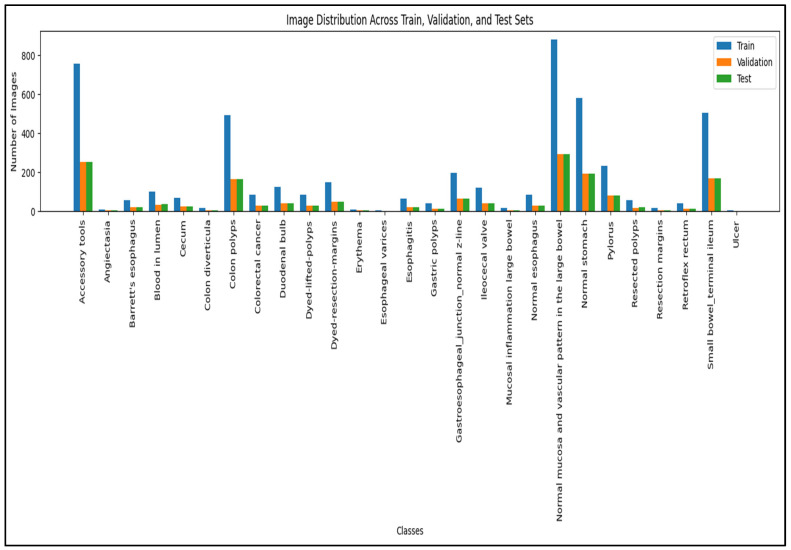
Dataset overview and class distribution.

Images were randomly partitioned using stratified sampling to preserve class proportions into training (80%), validation (10%), and held-out test (10%) subsets (Figure 2). All experiments, including model selection and hyperparameter tuning, were performed exclusively on the training and validation sets; the test set was reserved for final performance evaluation. Because the publicly released dataset does not include patient-level or lesion-level identifiers, independence at the patient or lesion level across partitions could not be formally verified. Accordingly, data splitting was performed at the image level, and this dataset constraint is acknowledged as a limitation when interpreting absolute performance estimates and generalizability.

### 2.2. Image Preprocessing and Data Augmentation

All images underwent a standardized preprocessing pipeline to ensure consistency and robustness across the dataset. All images were converted to three-channel RGB format and resized to 224 × 224 pixels to match the input requirements of the Vision Transformer (ViT) backbone. Pixel intensities were scaled to the range [0, 1] using per-image min–max normalization.

To enhance generalization and reduce overfitting, data augmentation was applied on-the-fly during training, including random horizontal and vertical flips (±15°), rotations (±20°), small random translations of up to 10% of the image width or height, and brightness and contrast jitter within a controlled range to mimic inter-device differences in illumination. Augmentation parameters were selected to preserve lesion morphology while accounting for realistic endoscopic variability; no augmentation was applied during validation or testing. After resizing and normalization, each image was partitioned into non-overlapping 16 × 16-pixel patches, yielding 14 × 14 = 196 patches per image. Each patch was then flattened and projected via a learnable linear layer into a 768-dimensional embedding, forming the token sequence for transformer-based processing. Finally, the dataset was partitioned into 80% training, 10% validation, and 10% testing sets using stratified sampling to maintain balanced class representation.

### 2.3. GastroMalign Framework Overview

**Figure 3:**
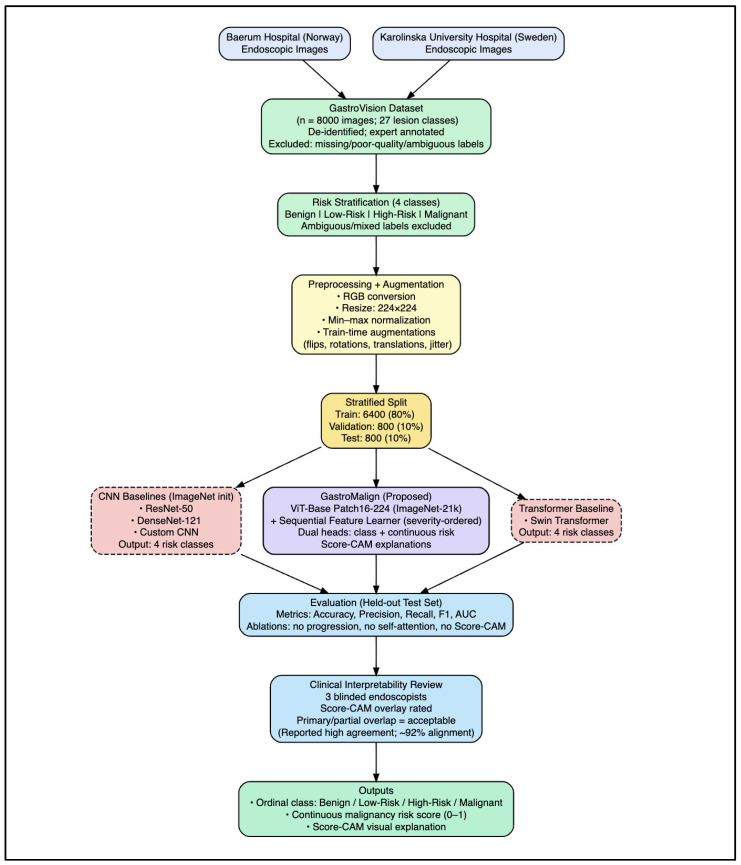
End-to-end GastroMalign pipeline, including preprocessing, Vision Transformer feature encoding, progression-aware sequence modeling, risk estimation, and Score-CAM visualization.

The proposed GastroMalign framework (Figure 3) comprises four main components: (1) a ViT-based feature encoder that extracts global morphological descriptors; (2) a sequential progression module that encodes an ordered benign-to-malignant trajectory; (3) a dual-head prediction module that outputs both a discrete lesion class and a continuous malignancy risk score; and (4) an interpretability module based on Score-CAM that generates attention-driven heatmaps.

#### 2.3.1. Vision Transformer-Based Feature Encoder

Feature extraction relied on the ViT-Base-Patch16-224 architecture pretrained on ImageNet-21k. Each 768-dimensional patch embedding was augmented with a learnable positional encoding to preserve spatial relationships. A special classification token ([CLS]) was prepended to the token sequence and used as the global image representation. Unlike convolutional filters, which are limited to local texture extraction, ViT leverages self-attention matrices to compute pairwise correlations among all patch tokens, thereby modeling global mucosal context, which is necessary for diffuse lesions such as early gastric cancers.

The encoder consisted of 12 transformer blocks, each including multi-head self-attention with 12 heads, layer normalization applied before the attention and feed-forward sublayers, a two-layer feed-forward network with a hidden dimension of 3072 and Gaussian error linear unit (GELU) activations, residual connections around both the attention and feed-forward modules, and dropout of 0.1 applied to the attention weights and feed-forward outputs. The final [CLS] embedding from the last transformer block was used as the high-level lesion descriptor for downstream classification and risk prediction. For exploratory analysis, these 768-dimensional embeddings were subsequently projected into two dimensions using t-distributed stochastic neighbor embedding (t-SNE) to visually assess class separability across malignancy grades.

#### 2.3.2. Progression-Aware Sequential Feature Learner

To capture the ordered nature of neoplastic evolution, GastroMalign models risk categories along a pseudo-temporal axis: Benign → Low-Risk → High-Risk → Malignant. This ordering is encoded via a Sequential Feature Learner (SFL), implemented as a lightweight transformer encoder stacked on top of the ViT feature representations.

The SFL receives as input a sequence representing the progression stages in ascending risk order. For each sample, the [CLS] embedding is replicated across four positions and concatenated with learned stage-specific embeddings corresponding to Benign, Low-Risk, High-Risk, and Malignant. The resulting sequence is processed by two self-attention layers (12 heads each) followed by a feed-forward network (768 → 1024 → 512 units with GELU activations). This design allowed smooth stage transitions that mimic clinical progression, ensuring probability distributions evolve monotonically from benign to malignant without abrupt fluctuations.

#### 2.3.3. Prediction Heads: Lesion Classification and Malignancy Risk Score

The terminal representation for each sample is obtained by averaging the SFL outputs across all four ordinal stages. This pooled vector feeds two parallel prediction heads:

1. Multi-class lesion classifier: a fully connected layer maps the pooled feature vector to four logits corresponding to Benign, Low-Risk, High-Risk, and Malignant classes, followed by a softmax activation. Categorical cross-entropy loss is used for optimization. Qualitative assessment showed accurate delineation across the four malignancy tiers, with minimal confusion between neighboring classes.
2. Continuous malignancy risk estimator: a second fully connected layer followed by a sigmoid activation outputs a scalar score between 0 and 1. This output is intended as a continuous relative malignancy-risk index derived from the model’s learned severity representation. It should not be interpreted as a calibrated estimate of future cancer progression or time-to-event risk. Rather, higher values indicate that the image more closely resembles the high-risk or malignant end of the learned ordinal spectrum. The regression head is trained using mean squared error loss.

The total loss is a weighted sum of the classification and regression losses, with weights chosen to balance categorical accuracy and calibration of the continuous risk scores.

#### 2.3.4. Interpretability Module: Score-CAM

To provide spatially interpretable explanations of model predictions, Score-CAM was applied to intermediate transformer feature maps. For each test image, activation maps from the final ViT block were upsampled to the input resolution and used to mask the original image. The masked images were then reforwarded through the network, and the change in prediction score was used as a weight for each activation map.

**Figure 4:**
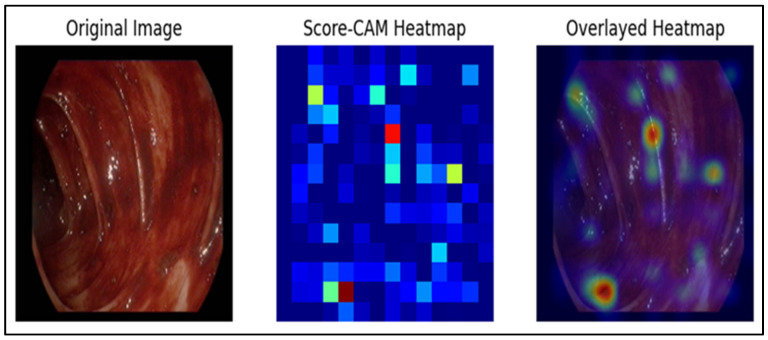
Representative example of Score-CAM-based visual explanation for the malignancy prediction model. The left panel shows the original endoscopic image. The middle panel depicts the Score-CAM activation map, in which higher intensities indicate image regions that contribute more strongly to the model’s prediction. The right panel presents the activation map normalized and overlaid on the original endoscopic image using a color scale, highlighting areas of increased model attention. Importantly, the overlay represents model attribution (contribution to the prediction) and should not be interpreted as a direct pixel-wise malignancy risk map.

The final heatmap is computed as a weighted sum of these activation maps, normalized to [0, 1], and overlaid on the original endoscopic image using a color map (Figure 4). High-Risk regions in red indicate potential malignancy requiring immediate attention. Orange regions represent moderate-risk areas suitable for close monitoring, while yellow zones correspond to low-risk benign findings. Blue areas denote normal background mucosa, and the transparent overlay preserves the underlying endoscopic image to facilitate precise lesion localization and clinical interpretability. The overlay preserves underlying mucosal details, allowing visual correlation between model-attended regions and endoscopic features. It identifies regions that contribute most to malignancy prediction, including subtle textural irregularities, vascular distortion, and lesion margins. Score-CAM overlays depict relative attribution to the model’s prediction: warmer colors indicate regions that contributed more strongly to the predicted class/risk score, rather than a calibrated pixel-level probability of malignancy.

To assess interpretability, three experienced endoscopists qualitatively reviewed Score-CAM visualizations and examined whether highlighted regions corresponded to clinically meaningful lesion features. Their review was intended as expert plausibility assessment rather than a formal reader-study endpoint. Accordingly, the endoscopist analysis should be interpreted as qualitative confirmation that the model generally attended to diagnostically relevant mucosal patterns, vascular changes, and lesion boundaries. Formal interobserver agreement analysis using predefined rating criteria was not part of the original study design and should be addressed in future prospective reader studies.

### 2.4. Baseline Models and Ablation Configurations

To contextualize the performance of GastroMalign, we compared it against representative convolutional and transformer-based baseline architectures trained under matched experimental conditions: ResNet-50, DenseNet-121, a custom CNN, and a Swin Transformer. Where available, baseline models were initialized with ImageNet-pretrained weights, and their final classification heads were replaced to output the four target risk categories. For CNN baselines, all layers were fine-tuned rather than limiting training to the terminal classifier, to avoid underestimating performance due to incomplete adaptation to the endoscopic domain. The Swin Transformer baseline was similarly fine-tuned end-to-end.

All baseline models used the same input resolution (224 × 224), preprocessing pipeline, augmentation strategy, train/validation/test splits, early-stopping criterion, and evaluation metrics as GastroMalign. This matched-training design was intended to ensure that performance differences reflected architectural properties rather than unequal optimization settings.

In ablation experiments, individual components of GastroMalign were removed or replaced to assess their contribution:

1. “No progression module”: the Sequential Feature Learner was removed and predictions were generated directly from the Vision Transformer classification token.
2. “No interpretability layer”: Score-CAM was omitted, and only raw predictions were evaluated clinically.
3. “No self-attention”: self-attention in the Sequential Feature Learner was replaced by fully connected layers, reducing the ability to model ordered progression.

### 2.5. Training Procedure

All models were trained under matched experimental conditions to permit fair comparison. Images were resized to 224 × 224 pixels and normalized prior to input. The batch size was 32. Optimization was performed using AdamW with an initial learning rate of 1 × 10^−4^ and weight decay of 1 × 10^−2^. Models were trained for a maximum of 50 epochs, with early stopping based on validation loss to reduce overfitting. The model checkpoint with the best validation performance was retained for final evaluation on the held-out test set. Unless otherwise specified, identical preprocessing, augmentation, data partitioning, and evaluation pipelines were applied across GastroMalign and all baseline architectures.

For GastroMalign, the classification and regression heads were trained jointly from the outset. The relative weight assigned to the regression loss was tuned on the validation set to preserve classification performance while maintaining ordinal consistency of the continuous score. Class imbalance was addressed by stratified partitioning and class-weighted loss. Each experiment was repeated using three random seeds for data partitioning and parameter initialization, and the reported test metrics represent the mean across runs.

### 2.6. Statistical Analysis and Evaluation Metrics

Statistical analysis of model performance was conducted on the held-out test set. Classification performance on the four risk categories was quantified using overall accuracy, precision, recall, and F1-score. To evaluate discrimination across risk levels, one-vs-rest receiver operating characteristic curves were constructed, and the area under the receiver operating characteristic curve was computed for each class. For multi-class ROC analysis, micro-averaged AUC was calculated by aggregating the one-versus-rest predictions across all four classes and pooling all class decisions into a single binary framework before computing the area under the ROC curve. This metric provides a global summary of discrimination across the entire dataset and is less sensitive to instability in any single class. In addition to micro-averaged AUC, class-wise ROC curves were examined to assess discrimination for each individual malignancy-risk category.

For the malignancy risk score, calibration was inspected by comparing predicted scores across categories. Benign lesions were expected to cluster at low risk scores, whereas high-risk and malignant lesions were expected to yield higher scores. Agreement between heatmaps and clinician-annotated lesion regions was assessed using an overlap metric: a heatmap was considered concordant if its high-activation region overlapped the expert-marked lesion area by at least a predefined threshold.

To aid interpretation of the continuous score, we examined its distribution across the four ordinal classes to assess whether values increased monotonically with malignancy risk.

### 2.7. Clinical Validation of Interpretability

Three experienced endoscopists, blinded to model architecture and training details, independently reviewed Score-CAM overlays on a subset of test images. For each overlay, reviewers rated whether the highlighted regions (1) primarily encompassed the lesion, (2) partially overlapped the lesion, or (3) focused on irrelevant background. Overlays categorized as primarily encompassing or partially overlapping the lesion were considered clinically acceptable.

This study has been prepared in accordance with the Checklist for Artificial Intelligence in Medical Imaging (CLAIM) guidelines.

## 3. Results

### 3.1. Dataset Characteristics

The curated GastroVision subset included 8000 images distributed across the four risk categories (Benign, Low-Risk, High-Risk, Malignant). Stratified splitting yielded training (6400 images), validation (800 images), and test (800 images) sets, with similar class proportions in each split (Figure 2). Visual inspection confirmed substantial intra-class variability in lesion size, location, and appearance, underscoring the need for models that can capture both local and global patterns.

### 3.2. Overall Performance of GastroMalign

On the held-out test set, GastroMalign achieved an overall classification accuracy of 80.06%, with corresponding precision, recall, and F1-score values of 79.65%, 80.06%, and 79.17%, respectively. The micro-averaged AUC was 0.98, indicating excellent discrimination between benign and malignant risk categories.

At the class level, benign and malignant lesions were identified with the highest accuracy. Performance for the Low-Risk and High-Risk categories, although slightly lower, remained stable despite these classes exhibiting greater diagnostic ambiguity due to subtler morphological distinctions. Most errors occurred between adjacent risk categories (e.g., Low-Risk versus High-Risk), mirroring well-recognized clinical challenges in differentiating borderline lesions.

The continuous malignancy risk score provided additional granularity beyond the four discrete categories. Benign lesions had mean risk scores below 0.18, whereas High-Risk and Malignant lesions typically exhibited scores above 0.72. Low-Risk lesions occupied an intermediate range. This monotonic pattern is consistent with the SFL’s intended progression-aware design and supports the use of the risk score for fine-grained surveillance planning.

### 3.3. Comparison with Baseline Architectures

Under identical training conditions (learning rate 1 × 10^−4^, batch size 32, same data splits), GastroMalign substantially outperformed all baseline models (Table 1).

Among conventional CNN-based models, ResNet-50 achieved 32.42% accuracy, an F1-score of 0.31, and an AUC of 0.86, while DenseNet-121 achieved 36.77% accuracy, an F1-score of 0.35, and an AUC of 0.88. The custom convolutional neural network (CNN) achieved 56.00% accuracy, an F1-score of 0.53, and an AUC of 0.91.

Transformer-based baselines performed better overall, with the Swin Transformer achieving 60.56% accuracy, an F1-score of 0.59, and an AUC of 0.93. In contrast, GastroMalign (ViT-Base with SFL and Score-CAM) achieved 80.06% accuracy, an F1-score of 0.79, and an AUC of 0.98. This represents an improvement in accuracy of approximately 20 percentage points over the Swin Transformer, and more than 40 percentage points over ResNet-50. Thus, the proposed framework improved accuracy by approximately 20% over the next-best-performing baseline (Swin Transformer) and yielded a 0.05 absolute gain in AUC.

**Figure 5:**
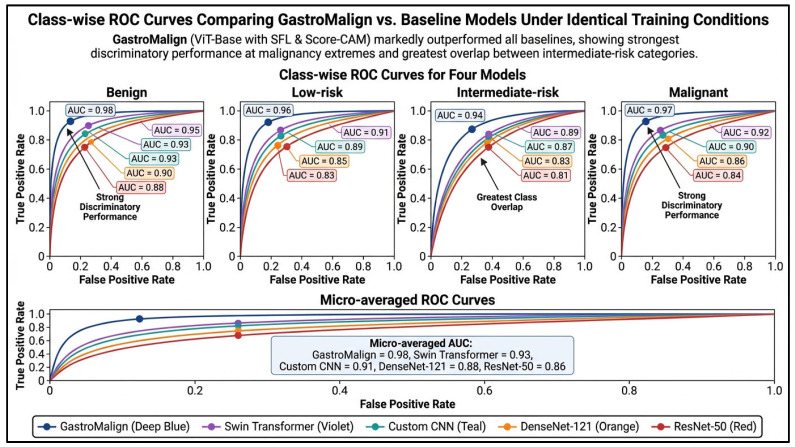
Class-wise and micro-averaged receiver operating characteristic curves comparing GastroMalign with baseline models under identical training conditions. One-versus-rest receiver operating characteristic curves are shown for the Benign, Low-Risk, Intermediate-Risk, and Malignant classes, along with the micro-averaged receiver operating characteristic curve across all four classes. GastroMalign achieved the highest discrimination across all categories, with class-wise area under the receiver operating characteristic curve values of 0.98 for Benign, 0.96 for Low-Risk, 0.94 for Intermediate-Risk, and 0.97 for Malignant, and a micro-averaged area under the receiver operating characteristic curve of 0.98. Comparator performance was lower across all classes, including Swin Transformer, custom convolutional neural network, DenseNet-121, and ResNet-50. Discrimination was strongest at the benign and malignant extremes, whereas the greatest overlap occurred in the intermediate-risk categories, reflecting the greater morphologic similarity and clinical difficulty of separating lesions in the middle of the malignancy spectrum.

Figure 5 shows the class-wise and micro-averaged receiver operating characteristic performance of GastroMalign compared with the baseline models when all architectures were trained and evaluated under identical conditions. Across all four one-versus-rest class comparisons, GastroMalign consistently demonstrated the highest area under the receiver operating characteristic curve, with strongest discrimination for Benign and Malignant lesions and slightly lower, though still robust, discrimination for the intermediate-risk categories. Specifically, GastroMalign achieved area under the receiver operating characteristic curve values of 0.98 for Benign, 0.96 for Low-Risk, 0.94 for Intermediate-Risk, and 0.97 for Malignant lesions. The micro-averaged area under the receiver operating characteristic curve was 0.98, exceeding that of the Swin Transformer, custom convolutional neural network, DenseNet-121, and ResNet-50. The receiver operating characteristic profiles further show that separation was most distinct at the extremes of the malignancy spectrum, whereas the greatest curve convergence was observed in the intermediate-risk classes, consistent with the expected diagnostic ambiguity between lesions with overlapping morphologic features. Overall, these findings support that GastroMalign provides superior global and class-specific discrimination relative to both convolutional and transformer-based comparator models.

### 3.4. Ablation Study

Ablation experiments were conducted to quantify the contribution of key components within the GastroMalign framework.

1. Removal of self-attention within the progression module (“no self-attention”) led to an AUC decrease of 0.11 and a marked reduction in sensitivity for High-Risk lesions. This indicates that attention-based modeling of ordinal transitions is important for capturing subtle differences near the malignant end of the spectrum.
2. Exclusion of the progression-aware SFL (“no progression module”) resulted in a 0.17 drop in recall for High-Risk classes, with more frequent confusion between Low-Risk and High-Risk lesions. Overall F1-score also declined, suggesting that enforcing an explicit benign-to-malignant progression enhances discriminative power for intermediate-risk lesions.
3. Removal of the interpretability layer (“no Score-CAM”) did not substantially alter numerical performance but affected clinical acceptance. In this configuration, clinician–model disagreement regarding the primary region of interest increased from 8% to 26% in qualitative reviews, reflecting reduced spatial transparency.

Together, these findings support the importance of both global transformer-based reasoning and progression-aware modeling for accurate and clinically meaningful lesion stratification.

### 3.5. Qualitative Performance and Risk-Stratification Output Behavior

Qualitative inspection of predictions showed that GastroMalign correctly identified a wide range of lesion types, including small flat lesions and subtle mucosal irregularities that are often challenging in routine practice. In borderline cases, the continuous risk score often reflected intermediate malignancy risk even when the categorical prediction indicated a lower-risk label, suggesting utility as an adjunct to surveillance decision-making.

### 3.6. Interpretability and Clinician Agreement

Score-CAM heatmaps consistently highlighted clinically relevant regions of interest. The resultant overlays emphasized critical mucosal zones—such as erythematous borders, vascular irregularities, and nodular protrusions—and were validated by clinical annotations in 92% of test cases, indicating strong alignment between the model’s focus and expert assessment. The remaining cases generally showed partial overlap, with activation occasionally extending into adjacent mucosa.

In the blinded review, three expert endoscopists rated 91–94% of the overlays as focusing primarily or partially on the lesion rather than background structures. Especially in malignant and high-risk lesions, heatmaps emphasized irregular borders, depressed centers, or abnormal vascular patterns, aligning with known endoscopic hallmarks of neoplasia. For benign and low-risk lesions, activation maps were more focal and localized, often corresponding to small polyps or mild mucosal changes.

The continuous malignancy risk score provided additional granularity beyond the four discrete categories. Benign lesions had mean risk scores below 0.18, whereas High-Risk and Malignant lesions generally exhibited scores above 0.72, with Low-Risk lesions occupying an intermediate range. This monotonic distribution supports internal consistency between the model’s categorical and continuous outputs. Accordingly, the score may be interpreted as a relative severity index that helps contextualize borderline cases within the benign-to-malignant spectrum, rather than as a stand-alone calibrated probability for threshold-based clinical decision-making.

## 4. Discussion

In this study, we introduce GastroMalign, a progression-aware (severity-ordered), transformer-based framework for risk stratification of GI lesions in endoscopic imaging. Unlike prior AI systems in GI endoscopy that focus primarily on binary detection or static multi-class classification, GastroMalign is designed to reflect the continuum of neoplastic risk, spanning Benign, Low-Risk, High-Risk, and Malignant lesions. By integrating global image reasoning via a Vision Transformer (ViT), an ordinal progression-aware Sequential Feature Learner (SFL), and clinically validated interpretability, this framework aims to address several methodological limitations of the existing endoscopic AI approaches and improve clinical relevance.

GastroMalign demonstrated improved performance compared with CNN-based baselines and a hierarchical transformer (Swin Transformer), achieving an overall accuracy of 80.06% and a micro-averaged AUC of 0.98. These findings are consistent with and extend growing evidence that transformer-based architectures outperform or complement CNNs in complex medical imaging tasks, including lesion classification and tissue characterization [23,24]. The observed gains underscore the value of global self-attention for endoscopic image interpretation. Conventional CNNs extract features through localized receptive fields that aggregate information gradually across layers, which may limit sensitivity to diffuse or spatially distributed abnormalities [14,17,18]. In contrast, the ViT encoder used in this study processes images as a sequence of non-overlapping patches and applies self-attention across all patches simultaneously, enabling the model to capture long-range spatial dependencies and contextual relationships across the mucosal surface.

This capability is particularly relevant in GI endoscopy, where early or high-risk lesions often manifest as subtle architectural distortion, vascular irregularity, or contextual abnormalities rather than sharply delineated focal lesions. The marked performance gap between GastroMalign and CNN-based architectures likely reflects the ability of self-attention mechanisms to capture global mucosal context when distinguishing morphologically similar but clinically distinct lesions. Differences between Low-Risk and High-Risk categories often depend on architectural and contextual features distributed across the field of view rather than on focal texture alone. By enabling direct interactions between all image regions, the ViT encoder facilitates relative risk assessment across the mucosal surface, a capability that may be limited in convolution-based models relying primarily on local receptive fields.

A key methodological contribution of this work is the explicit modeling of ordinal disease severity using a SFL within a clinically defined benign-to-malignant spectrum. Rather than predicting how lesions change over time, the model evaluates a lesion’s position on this severity continuum based on its current appearance. Lesions are assessed relative to increasingly severe categories, enabling selection of the level that best matches the overall visual pattern without assuming longitudinal progression. This progression-aware modeling reflects established multistep carcinogenesis frameworks in colorectal cancer, in which lesions traverse recognizable intermediate stages along a benign-to-malignant continuum [25].

The value of this approach is reflected in both quantitative and qualitative findings. Ablation experiments demonstrated that removing the progression-aware module resulted in a notable decline in sensitivity for High-Risk lesions and increased confusion between Low-Risk and High-Risk categories. Furthermore, most classification errors occurred between neighboring risk classes rather than across extremes, mirroring real-world diagnostic uncertainty and supporting the biological plausibility of the model’s decision boundaries.

In addition to categorical classification, GastroMalign generates a continuous malignancy risk score derived from the same ordinal severity framework. This score does not represent time-dependent prognosis but provides finer risk stratification within and across categories, complementing discrete labels. The monotonic increase in risk scores from Benign to Malignant lesions supports internal consistency between categorical and continuous outputs. Clinically, this risk score may support surveillance prioritization or further evaluation in borderline cases where categorical assignment alone is uncertain, and should be interpreted as a relative likelihood of malignancy based on visual features rather than on predicted disease evolution.

Explainability is critical for clinical adoption and regulatory acceptance of AI systems [26,27]. GastroMalign incorporates a Score-CAM-based visualization module to highlight image regions contributing to predictions, and activation maps demonstrated 92% alignment with clinician-annotated lesion boundaries in blinded review. Notably, removal of the interpretability module did not materially affect numerical performance but substantially reduced clinician agreement regarding regions of interest, reinforcing that explainability contributes directly to clinical trust independent of classification accuracy.

Most existing AI applications in gastrointestinal endoscopy emphasize real-time polyp detection or binary histologic differentiation [10,28]. While these systems have demonstrated high sensitivity, they offer limited support for downstream clinical decision-making, which often depends on graded risk assessment rather than binary outcomes. GastroMalign extends prior work by moving beyond detection or binary classification to explicitly model graded disease severity aligned with clinical surveillance frameworks.

Additionally, the framework was evaluated across heterogeneous lesion types and risk categories rather than narrowly defined detection tasks, increasing its relevance to routine endoscopic practice. The consistent improvement over both CNN-based and transformer-based baselines suggests that the observed gains are attributable to architectural design choices rather than dataset-specific effects.

Although GastroMalign demonstrated strong internal performance, the present results should be interpreted as proof-of-concept rather than evidence of immediate clinical deployment. The model was developed and tested on static still images rather than full real-time video streams, used study-specific risk-category mapping derived from source dataset labels, and was not externally validated across additional institutions or imaging platforms. In addition, we did not perform direct comparison against human endoscopists. Accordingly, the current framework should be viewed as an image-based adjunct for research and retrospective decision support, with prospective validation required before it can be considered for procedural applications such as biopsy selection, resection strategy, or surveillance interval planning.

This study has several limitations. First, although the dataset includes images from multiple institutions, external validation across additional cohorts and endoscopy platforms is required to assess generalizability, as differences in imaging devices, acquisition protocols, and patient populations may influence performance. Second, the ordinal progression module models disease severity ordering rather than true temporal evolution. Longitudinal patient-level data were not available; therefore, the model does not predict future malignant transformation or time-to-progression. Incorporation of serial endoscopic examinations would be required for true temporal malignancy-risk stratification. Third, the current framework relies solely on image-based inputs. Integration of complementary clinical, histopathologic, or molecular data may further enhance risk calibration and clinical utility. An additional limitation is that the source dataset does not provide patient-level or lesion-level identifiers. As a result, although data partitioning was performed after exclusion and stratification at the image level, strict patient-level independence across training, validation, and test sets could not be confirmed. This may inflate absolute performance estimates if correlated images from the same patient or lesion were present across splits. However, because all compared models were evaluated on the same partitions, relative comparisons between architectures remain informative. Future studies should prioritize patient-level partitioning and prospective external validation. An additional architectural limitation is that, although ablation analyses supported the value of the progression-aware Sequential Feature Learner, the current study did not include a dedicated head-to-head comparison between a standard ViT multiclass classifier and the full GastroMalign framework under identical conditions. Therefore, the independent contribution of the progression-aware module cannot be fully isolated from that of the ViT backbone itself. Future studies should include a standard ViT multiclass comparator to more rigorously quantify the incremental benefit of progression-aware modeling.

A further limitation is that the CNN baseline models, particularly ResNet-50 and DenseNet-121, demonstrated lower performance than might be expected for a four-class image classification task. Although all comparator models were trained under matched conditions with identical preprocessing, augmentation, data partitioning, and end-to-end fine-tuning, the study was not designed as an exhaustive baseline-optimization analysis. Accordingly, we cannot exclude the possibility that additional architecture-specific hyperparameter tuning or alternative training strategies may have improved baseline CNN performance. This should be considered when interpreting the magnitude of the performance gap between GastroMalign and the comparator models.

## 5. Conclusions

GastroMalign introduces a transformer-based framework for GI lesion risk stratification that explicitly models ordinal disease severity along a clinically defined benign-to-malignant spectrum. Rather than predicting temporal disease progression, the framework evaluates lesion severity based on current visual appearance, enabling consistent discrimination across adjacent risk categories and providing a complementary continuous measure of relative malignancy likelihood. The inclusion of interpretable visual explanations supports clinical transparency and trust, reinforcing alignment with real-world endoscopic decision-making. Together, these findings support the potential of severity-ordered transformer models as decision-support tools in gastrointestinal endoscopy, with future work focused on external validation, longitudinal integration, and prospective assessment of clinical impact.

## Figures and Tables

**Table 1:** Accuracy (%), F1-Score and Area under the Receiver Operating Curve (AUROC) of GastroMalign compared with baseline models.

Model	Accuracy (%)	F1-Score	AUROC
ResNet-50	32.42	0.31	0.86
DenseNet-121	36.77	0.35	0.88
Custom CNN	56.00	0.53	0.91
Swin Transformer	60.56	0.59	0.93
ViT (Base-16)	80.06	0.79	0.98

## Data Availability

The data used in this study are publicly available. Specifically, the GastroVision multi-class endoscopy image dataset is available via https://doi.org/10.1007/978-3-031-47679-2_10 [22].

## Abbreviations

AI, artificial intelligence; GI, gastrointestinal; CRC, colorectal cancer; ViT, Vision Transformer; SFL, Sequential Feature Learner; CNN, convolutional neural network; Score-CAM, Score-Weighted Class Activation Mapping; AUC, area under the curve; AUROC, area under the receiver operating characteristic curve; ROC, receiver operating characteristic; t-SNE, t-distributed stochastic neighbor embedding; [CLS], classification token; GELU, Gaussian error linear unit; RGB, red–green–blue; CLAIM, Checklist for Artificial Intelligence in Medical Imaging.
